# Development and optimization of dapagliflozin oral nano-bilosomes using response surface method: *in vitro* evaluation, *in vivo* evaluation

**DOI:** 10.7150/ntno.99271

**Published:** 2025-01-01

**Authors:** Ananda Kumar Chettupalli, Nihar Ranjan Kar, V T Iswariya, Uttam Prasad Panigrahy, Laliteshwar Pratap Singh, Harekrishna Roy, Deepadarshan Urs, Muralidharan V, Sandhya Rani Mandadi, M Akiful Haque, Ritesh Rana, Talha Bin Emran

**Affiliations:** 1 Department of Pharmaceutical Siences, School of Pharmacy, Galgotias University, Greater Noida, Uttar Pradesh 203201, India.; 2 Centurion University of Technology and Management, Odisha, India.; 3 CMR College of Pharmacy, Kandlakoya, Medchal, Telangana, India.; 4 Faculty of Pharmaceutical Science, Assam Down Town University, Sankar Madhab Path, Gandhi Nagar, Panikhaiti, Guwahati, Assam, 781026, India.; 5 Narayan Institute of Pharmacy, Gopal Narayan Singh University, Sasaram, Bihar, India.; 6 Department of Pharmaceutics, Nirmala College of Pharmacy, Mangalagiri, Guntur, Andhra Pradesh, India.; 7 Inflammation Research Laboratory, Department of Studies and Research in Biochemistry, Mangalore University, Karnataka, India.; 8 Vishnu Institute of Pharmaceutical Education and Research, Narsapur, Telangana, India.; 9 Himachal Institute of Pharmaceutical Education and Research, Nadaun, Himachal Pradesh, India.; 10 Department of Pathology and Laboratory Medicine, Warren Alpert Medical School, Brown University, Providence, RI 02912, USA.; 11 Legorreta Cancer Center, Brown University, Providence, RI 02912, USA.; 12 Department of Pharmacy, Faculty of Allied Health Sciences, Daffodil International University, Dhaka 1207, Bangladesh.

**Keywords:** Dapagliflozin, Nano Bilosomes, hyperglycemia, glucotoxicity, Permeation study, Edge activator

## Abstract

In treating type 2 diabetes, avoiding glucose reabsorption (glucotoxicity) and managing hyperglycemia are also important. A metabolic condition known as diabetes (type-2) is characterized by high blood sugar levels in comparison to normal Bilosomes (BLs) containing Dapagliflozin (Dapa) were formulated, optimized, and tested for oral therapeutic efficacy in the current investigation. Used the Box Behnken design to optimize the Dapa-BLs, formulated via a thin-film hydration technique. Bile salts (X1) concentration, edge activator (X2) in mg, and non-ionic surfactant (X3) were the independent variables. The Entrapment Efficiency (Y1), Particle size (PS), polydispersity index (PDI), and zeta potential (ZP), were selected as dependent variables. To get the optimal formula, use Design-Expert® software for numerical optimization. The optimal bilosomal formula was selected by boosting %EE, ZP (absolute value), and *in vitro* drug release while also considering decreasing PS and PDI. *Ex vivo* skin permeation, Fourier transform infrared spectroscopy (FTIR), differential scanning calorimetry (DSC), and scanning electron microscopy (SEM) were evaluate the optimized formulation. The *in vivo* pharmacodynamics activities of the optimized formula were examined on rats and compared to that of the oral Dapa solution. The optimized Dapa-BLs were shown a particle size of 155.36±2.48 nm and an entrapment efficiency of 86.37±2.6%. The SEM image showed a spherical particle with sharp boundaries. The drug release study revealed a significant enhancement in Dapa release (75.31 ± 2.68%) from Dapa -BLs as compared to drug solution (30.46 ± 3.64%). The results of the exvivo permeation and pharmacokinetic studies revealed a 4.49 times higher flux and 3.41 folds higher AUC_0-t_ than drug solution. The antidiabetic activity results showed significant (P < 0.05) enhancement in therapeutic efficacy than drug solution. The results also showed marked improvement in biochemical parameters. Our findings suggested, the prepared Dapa loaded bilosomes was found to be an efficient delivery in the therapeutic efficacy in diabetes.

## Introduction

Calcium-glucose cotransporter 2 (SGLT2) can be blocked very effectively, specifically, and reversibly with Dapagliflozin (Dapa). It functions by reducing the kidneys' absorption of glucose, leading to a rise in urine glucose excretion, and enhancing glycemic control in individuals with type 2 diabetes mellitus 1. Chemically speaking, Dapa is defined as (1S)-1,5-anhydro-1-C- [4-chloro-3- [(4-ethoxy phenyl) methyl] phenyl]. -D-glucito. It is a white, crystalline powder soluble in dimethylformamide, ethanol, methanol, and DMSO 2. The European Medicines Agency (EMA) categorizes Dapa as Class III in the Biopharmaceutics Classification System (BCS) due to its high solubility and limited permeability 3. The US Food and Drug Administration (FDA) approved the commercial formulation of this medication, Forxiga®, in January 2014. It was the first medication of its kind to receive approval from the European Union 4. Brazil's Health Surveillance Agency (ANVISA) gave its permission in July 2013 5, 6.

The most efficient method of administering medication is through oral ingestion, especially for individuals with long-term ailments. Although oral administration offers numerous benefits, certain drugs experience limited oral bioavailability. This could be linked to the first-pass metabolism process, the medication's restricted solubility and permeability, or drug efflux 7. The rate-limiting process of dissolution, which leads to inconsistent absorption, causes the reduced bioavailability of poorly soluble medications. Researchers have employed several strategies to increase the body's ability to absorb and utilize medications when taken orally 8, 9.

The prevalence of diabetes mellitus (DM) is on the rise as a result of inadequate nutrition and unhealthy lifestyle choices, leading to a substantial burden on individuals, families, and society 10. At present, DM management often involves the administration of insulin injections and oral antidiabetic drugs. Nevertheless, subcutaneous insulin injections can cause discomfort and are occasionally associated with an allergic reaction, lipodystrophy, hypoglycemia, and even hyperinsulinemia 11. Furthermore, oral antidiabetic drugs such as glipizide, metformin, and repaglinide may cause gastrointestinal adverse effects, pose a significant risk of hypoglycemia, and contribute to weight gain 12. These therapy methods may lessen some of the symptoms of DM, but they cannot fully cure the condition. New, efficient medications are still required to treat DM.

Given that they have various advantages over conventional dosage forms, there has recently been an increase in interest in researching nanocarriers for oral administration 13. These nanocarriers are also known to increase the rate at which poorly soluble medicines dissolve and successfully avoid first-pass metabolism by stimulating lymphatic transport, both of which boost bioavailability 14, 15. Researchers have established that the addition of charge-inducing substances and bile salts after oral delivery can influence the biological fate of vesicular systems 16. Bilosomes (BLs), an unorthodox colloidal dispersion, can manufacture and place bile salts (BS) within liposomes. BLs are nanoscale structures that contain bile salts and surfactants, indicating functional effectiveness in oral medication administration. According to Conacher *et al.* (2001) 17, the term "BL" refers to the newly developed platforms of nano-vesicular carriers for drug delivery. Increased liposomal resistance is a result of the presence of BS leads to increased liposomal resistance. When compared to regular liposomes, they are significantly more forgiving and adaptable. The lipid bilayers of BL, which contain bile salts, enhance their resistance to GI bile salts and enzymes, thereby protecting the entrapped vaccine from the harsh conditions of the GI tract. This makes them healthier and more effective than conventional vaccines. Additionally, they are noninvasive and diffraction-limited. The apical sodium-dependent bile acid transporter (ASBT) in the GIT also absorbs bile salts 18-20. This may make oral delivery of bile salts better. Using factorial designs is an excellent method for determining response and the interplay of numerous factors. Additionally, it facilitates numerical optimization, allowing for the discovery of the optimal formula based on the preferred conditions of each answer 21.

There are no known references to the use of bilosomes for enhancing Dapa transdermal distribution. To develop a sustained-release bilosomal system, the present study combined formula selection using the Box-Behnken design with numerical optimization and characterization. We subjected the optimized bilosomal formula to an *ex vivo* permeation investigation in order to assess the permeation characteristics of Dapa. We conducted a histological analysis and a skin irritancy test to confirm the safety of the produced bilosomes on human skin. Researchers were able to evaluate the effectiveness of the optimized bilosomal composition by examining its anti-inflammatory effect, antinociceptive activity, and *in vivo* penetration.

## Materials & Methods

### Materials

Purchasing Dapa from Zydus Cadila Ltd. (Ahmedabad, India). Sodium deoxycholate (SDC), sodium alginate (SA), cholesterol (CHO), Span 60, and cholesterol were purchased from SD Fine Chemical (Mumbai, India). Dialysis bags (MWCO 12 kDa), acetonitrile, chloroform, methanol, and chitosan (CH) were purchased from Sigma Aldrich (Bengaluru, India). Hi-media provided soya bean casein and fluid thioglycolate digest medium (Mumbai, India). Pluronic (P188), sodium cholate hydrate (SC), phosphatidylcholine (PDC), and phosphatidyl serine (PDS) were acquired from Sigma-Aldrich (Germany). The cholesterol (CH) source was CRODA Inc. (East Yorkshire, England). Analytical-grade materials were employed for all other compounds and reagents in this study.

### Preparation of Dapa loaded BLs

The Dapa-BLs was prepared with a slightly modified thin-film hydration method, reported by Wilkhu *et al.*, using Pluronic 123 (P123, 0.6%), cholesterol (CH, 0.3%), and edge activator (Tween 80). The detailed composition of Dapa-BLs is shown in Table 1. The ingredients were dissolved in an organic solvent (chloroform: methanol, 1:1). The mixture was transferred into a round-bottom flask and rotated in a rotary evaporator (Rotavapor, Heidolph VV 2000; Heidolph Instruments, Kehlheim, Germany) at 40 °C to evaporate the organic solvent under reduced pressure. A thin layer was formed on the surface of the round-bottom flask and was kept overnight in a desiccator to complete the removal of moisture. The film was hydrated for 1 h by taking a weighed quantity of SDC aqueous solution (10 mL) in a rotatory evaporator at 40 °C. Finally, the prepared Dapa-BLs dispersion was further ultrasonicated for 1 min/cycle at 30 s intervals to reduce the size.

### Experimental design

Used Box-Behnken design to optimize the Dapa-BLs utilizing three parameters and three levels 22, 23. Bile salt (sodium deoxy cholate (SDC), X1), an edge activator (Tween 80, B), and a surfactant (Span 60, C) were chosen as variables based on the preliminary study. As stated in Table 1, their impacts were seen on the following variables: entrapment efficiency (% as Y1), particle size (nm as Y2), PDI (Y3), and zeta potential (mV, Y4). We implemented a point prediction method to select the most optimized composition. To confirm the model's validity, the desirability value of each response is further assessed. We analyzed all the responses using polynomial equations and 3D response plots to establish the relationship between the independent factors and their corresponding answers. We analyzed the effects of independent variables on dependent variables using empirical models such as linear, second-order (2F1), and quadratic models. We independently calculated the regression coefficients of all the models and the analysis of variance (ANOVA) for the best-fit model.

### Entrapment Efficiency (EE %) of Dapa-BLs

Ultracentrifugation was employed to indirectly assess the effectiveness of Dapa trapping in bacterial lysates 24. The formulation underwent centrifugation for 90 min at 4°C and 14,000 revolutions per minute, with 1 mL of each solid-state liquid, in a cooling centrifuge. The supernatant was separated and analyzed for the drug using a UV spectrophotometer (Shimadzu, UV-2401 PC, Kyoto Japan) set to max = 235 nm (which corresponds to the calibration curve established using the provided Dapa wavelength) 25-27. Standard curves were generated in a phosphate citrate buffer with a pH of 6.8, and the regression equation was employed to accurately calculate the quantity of medication trapped compared to a blank. To calculate the EE%, we utilized the formula listed in a below Equation.

% EE = (DT-Ds)/DT X100

Theoretical dose (DT) of Dapa minus measured dose (DS).

### Particle Size (PS), Polydispersity Index (PDI) and Zeta Potential (ZP)

The initial steps involved diluting the dispersions to the correct concentrations with deionized water 29. Then, at (25±2°C) using a helium-neon laser at a wavelength of 633 nm, the mean ZP, PS, and PDI of the built equations were acquired by Zetasizer Nano ZS (Malvern Instruments, Malvern, UK). Three independent judges compared each formula.

### Characterization of the Optimized Bilosomal Formula

#### Differential Scanning Calorimetry (DSC)

Three to four milligrams of each sample of Dapa, SDC, Tween 80, cholesterol, p 123, and the optimized Dapa-BLs formula were heated thermograms were created by heating an aluminum pan to (350°C) in a nitrogen environment at a scanning rate of 10°C/min by a (DSC7, Perkin-Elmer, Waltham, MA, USA). 30.

#### Fourier Transform Infrared Spectroscopy Study (FTIR)

The Bilosome Approach to Formulation Using an FTIR Spectrophotometer (BRUKER-ALPHA, Specac, Germany), 31 we evaluated the various components of the improved Dapa-BLs formulation (F13). Just below the FTIR's fixed probe, 0.5 mL of the material was scanned over the wavenumber range of 3500-1000 cm^-1.^

#### Scanning electron microscopy

SEM was used to examine the manufactured particles' shape and size. Pushed Nanoparticles out of the chamber after being placed on a thin carbon sheet, where their dispersion patterns were studied using microscopy. The sample is scanned in rows as an intense primary electron beam is focused on a pinpoint using lenses. When the focused electron beam hits the surface, it ionizes the area and generates secondary electrons. A detector is used to tally the number of secondary electrons. An amplifier requires the collection of lateral electrons by a collector 32.

#### *In vitro* dissolution study

The dissolving analysis of the optimized Dapa-BLs (F13) and pure Dapa solution was conducted using a dialysis membrane 33. A dialysis membrane that had been pre-soaked was obtained. The tested samples, measuring 1 mL each, were then poured into test tubes. A test tube was secured by tying the dialysis bag to its opening. The experiment commenced by immersing the sealed bags into 100 mL of simulated intestinal fluid (SIF; pH = 6.8) at a controlled temperature of 37 ± 0.5 °C. The set underwent continuous agitation at a steady speed of 100 rpm using a magnetic stirrer. Samples of 2 mL were extracted at predetermined time intervals ranging from 0 to 24 hours (0, 0.5, 1, 1.5, 2, 4, 6, 8, 12, 16, 20, and 24 hours). The 100 mL STF release media was poured into a beaker at 37 ± 0.5 °C. The test tube was hung in the release medium, and the dissolution media was agitated at 50 revolutions per minute using a thermostat-controlled magnetic stirrer. At a specific time, 2 mL samples were collected from the beaker and replaced with fresh dissolving media to keep the volume constant during the investigation. The absorbance was measured using a UV-visible spectrophotometer at a wavelength of 235nm, and the drug release was determined using a Microsoft Excel spreadsheet 34, 35.

#### Permeation study

Animal ethical committee of Nalanda College of Pharmacy, Nalgonda, Telangana, authorized the work. The research followed the directives specified in the Guide for Care and Use of Laboratory Animals issued by the US National Institute of Health (NIH Publication No. 85-23, updated 2011). The enhancement in permeability of optimized Dapa-BLs and Dapa solution was evaluated by performing permeation study on the GIT of rats. To eliminate the meal contents, the rat intestine was retrieved and subsequently cleaned with water. A test sample comprising optimized Dapa-BLs and a Dapa solution (about 10 mg of Dapa) was inserted into an intestinal sac. The pouch was tightly sealed at both sides and then immersed in a 500 cc Phosphate buffer solution with a pH of 6.8, which closely mirrored the receptor medium. Periodically at predetermined time intervals (1, 2, 4, 6, 8, 10 hours), 0.5 mL samples were collected and immediately substituted with new receptor media to maintain a consistent concentration gradient. Employing an aerator guaranteed the supply of oxygen, while the temperature was continuously regulated at 37 ± 0.5 °C for the entire study. 2-mL samples were removed and substituted with an equivalent volume of fresh Phosphate buffer (pH=6.8) at specified time intervals. The collected samples were filtered and diluted, and the drug concentration at each time point was determined using the High-Performance Liquid Chromatography (HPLC) technique as described in reference 36-38. Measurements of permeation flux and apparent permeability were taken for both samples in order to assess the level of augmentation. A graph was generated to illustrate the rate of Dapa translocation across the membrane per unit area (µg/cm^2^) with respect to time (h). Quantification of Dapa per unit area across the membrane after 10 hours (Q10h), average flow rate after 10 hours (J_max_), improvement ratio (ER), and residual Dapa in the skin after 10 hours were assessed for both optimized Dapa-BLs and Dapa solution. All experiments were carried out in triplicate. The flux peak (J_max_) and the enhancement ratio (ER) were calculated using the following mathematical equations.

J_max_ = 
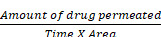


ER = 
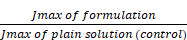


The flow parameters consist of the total measured amount of Dapa that has penetrated, the total time of the experiment, and the surface area of the membrane. Following the permeation study, the skin samples were thoroughly rinsed with 10 mL of normal saline solution in order to eliminate the adhering formulation. Furthermore, the samples were partitioned into smaller portions and exposed to sonication in a 50 mL phosphate buffer with a pH of 6.8 for a duration of 30 min. The objective of this procedure was to isolate the therapeutic substance that had gathered in the samples. The samples were subjected to centrifugation, and the liquid retained above the sediment was filtered using a 0.45 µm pore size filter. Quantification of the filtered liquid was performed using HPLC 39,40.

### Biological study

#### Animal handling

A preclinical study was conducted to evaluate the effects of Dapa-BLs and Dapa solution on Wistar albino rats weighing between 200 to 250 gm. The research received approval from the Institutional Animal Ethical Committee of Nalanda College of Pharmacy, Nalgonda, Telangana, under application No-I/IAEC/NCP/015/2022 WR. The animal was obtained from the animal breeding facility and maintained under controlled environmental conditions with a 12-hour dark/light cycle. The animals were provided with a conventional high-fat diet consisting of a normal pellet meal, cholesterol, casein protein, vitamins, coconut oil, sucrose, fructose, sodium chloride, and dl-methionine for a duration of 15 days. Determined each animal's blood glucose level (BGL) before and after treatment with Dapa-BLs and a placebo solution at the same dose using a glucometer (Accu-Check, Roche, Germany). The animals were split up into four groups:

- The normal controls (NC)
- The diabetic controls (DC)
- The drug solution (DS)
- The Optimized formulation of Dapa-BLs. Six rats make up each treatment group.

#### Induction of diabetes

Hyperglycemia (Type 2 diabetes) was induced in rats using a model consisting of streptozotocin (STZ) and injected with a high-fat diet, solitary low dose of STZ (35 mg/kg) (0.1 M) intraperitoneally. The STZ was newly produced in citrate buffer (pH 4.5). After letting the BGL stable for 72 hours, the animals' fasting BGL was tested. Animals classified as hyperglycemic were those with fasting blood glucose levels above 200 mg/dL and were excluded from further study 41, 42.

### Pharmacokinetic study

Tested Dapa-BLs and Dapa solution for pharmacokinetics in Wistar albino rats. Group 3 and 4 animals were given Dapa-BLs and a Dapa solution (10 mg/kg of Dapa) 43. Animals were given a diethyl ether inhaler to induce anesthesia and drew their blood into an EDTA tube at 0, 1, 2, 4, 8, 12, and 24 hours. The plasma was extracted from the blood after centrifuging at 5000 rpm for 15 min. The plasma was processed using a liquid phase extraction method to remove the Dapa. After combining formic acid (2% v/v) and plasma, the mixture was vortexed for 10 min. After that, 2 mL of ethyl acetate was added, and the mixture was centrifuged at 4 °C for 15 min (3000 rpm). The supernatant was evaporated with nitrogen after collection, and the therapeutic concentration was determined by injecting 20 L of the reconstituted sample into an HPLC system (Auto-sampler) via a 0.25 m membrane filter. To calculate pharmacokinetic parameters such as C_max_, T_max_, T_1/2_, AUC_0-t_, AUC_0-∞_, K_el_, and AUMC_0-24_, we used software (Excel add-on PK solver).

#### Evaluation of hyperglycemic activity

In groups 3 and 4, animals were administered Dapa-BLs and Dapa solution once daily at the appropriate dosage of 10 mg/kg using an oral feeding needle 44. Blood was drawn from the tail of rats at regular intervals using a glucometer strip. Within 5 seconds, the glucometer's display displayed the blood glucose level (BGL) reading. The reduction in BGL (%) was calculated using the given formula for both groups.

BGL reduction (%) = (BGL t-to-BGL t-t)/ (BGL t-to) X100

#### Biochemical evaluation

After the investigation was done, the biochemical variables were analyzed. After setting aside the blood sample for processing, the plasma was centrifuged at 3000 rpm for 15 min to separate the components. The blood plasma was chilled and kept in the fridge for biochemical analysis. The standard commercial kits to determine values for parameters such as lipid profile, creatinine, urea, serum glutamic-pyruvic transaminase, uric acid, total serum protein, and serum glutamic oxaloacetic transaminase by the PAP technique.

### Statistical analysis

Replicated the experiment three times, and the results are provided as the mean and standard deviation. Statistics were calculated using a graph pad prism. When the *p*< 0.05, the result is said to be significant.

## Results & Discussion

### Preparation of dapa-loaded BLs

Miere *et al.* (2021) stated the advantages of preparing liposomes using a mixture of phospholipids and cholesterol, as this combination proved to increase liposomal membrane permeability, and hence, increase the binding of liposomes with cells *in vivo*. Addition of bile salts to the aforementioned components to prepare BLs has proven to enhance drug solubility, stability, and permeation through gastrointestinal barriers. Bile salts are biocompatible with no toxicity profile. They can act as solubilizing and permeation-enhancing agent. Sodium deoxycholate (SDC) is one of the most commonly used bile salts due to its nontoxic nature and high permeation enhancing capacity and hence it was utilized to prepare Dapa BLs. The choice of P123 as a surfactant in the present study was based on several known advantages, including low immunogenicity, lack of irritation upon topical or subcutaneous application, and ready elimination by the kidneys. Moreover, the presence of polyethylene oxide groups in its structure can reduce BLs recognition by the mononuclear phagocytic system, further enhancing stability.

### Optimization

**Figure 1:**
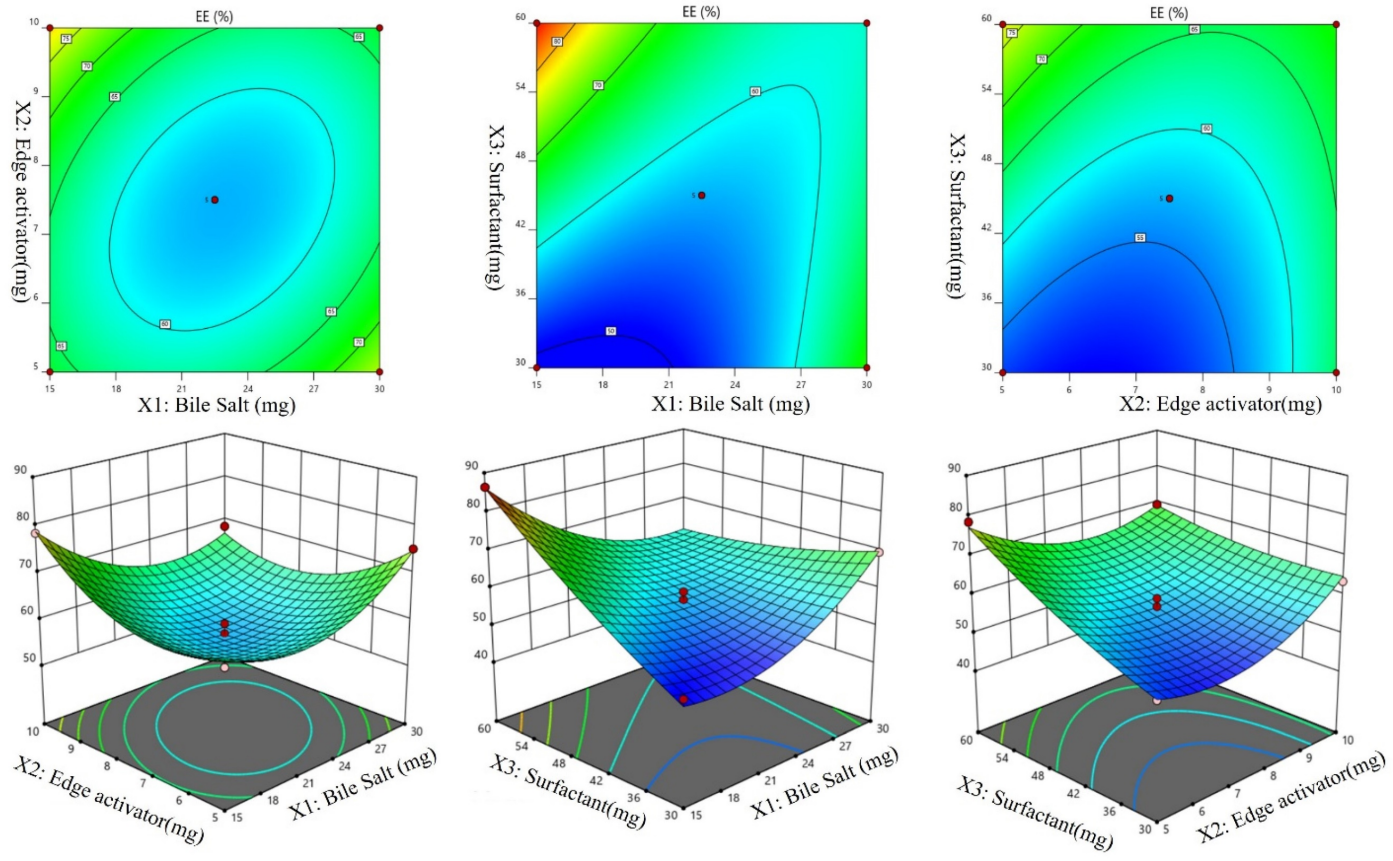
Effect of independent variables (A) Bile salts, (B) Edge activator, and (C) surfactant on entrapment efficiency (Y1).

**Figure 2:**
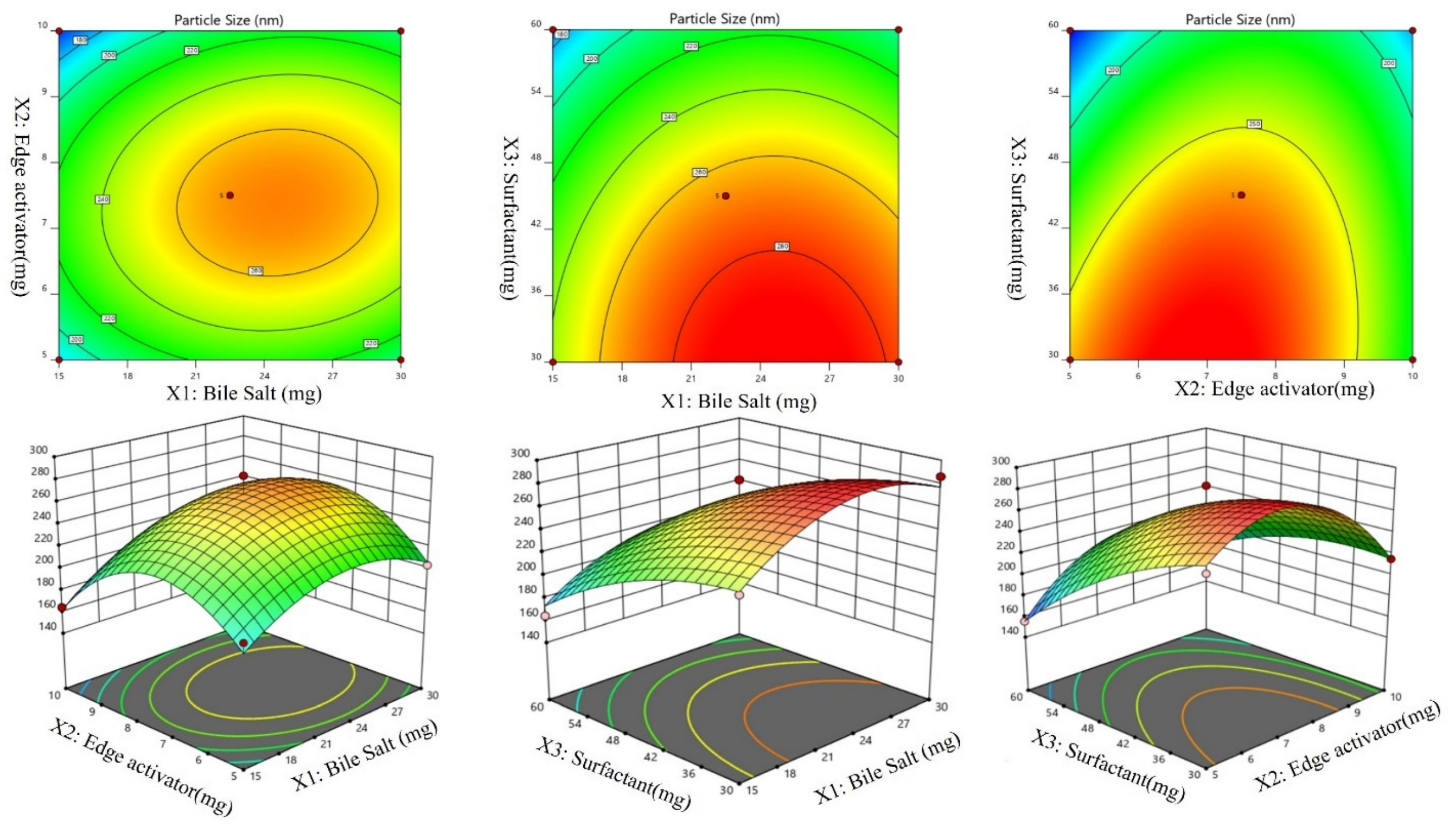
Effect of independent variables Bile salts (A), Edge activator (B), and Span 60 (C) on Particle size (Y2).

**Figure 3:**
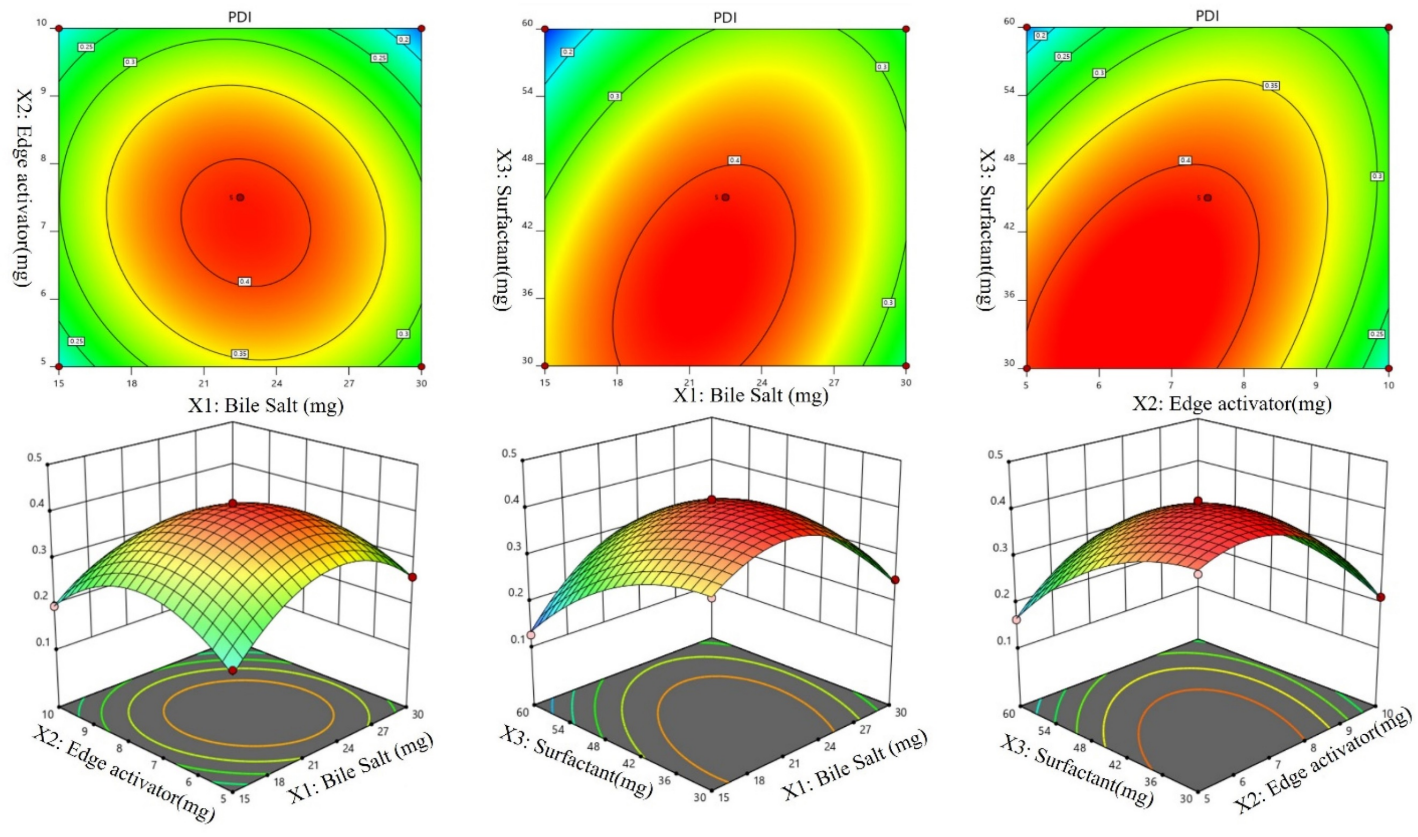
Effect of independent variables Bile salt (A), edge activator (B), and Span 60 (C) on PDI (Y_3_).

**Figure 4:**
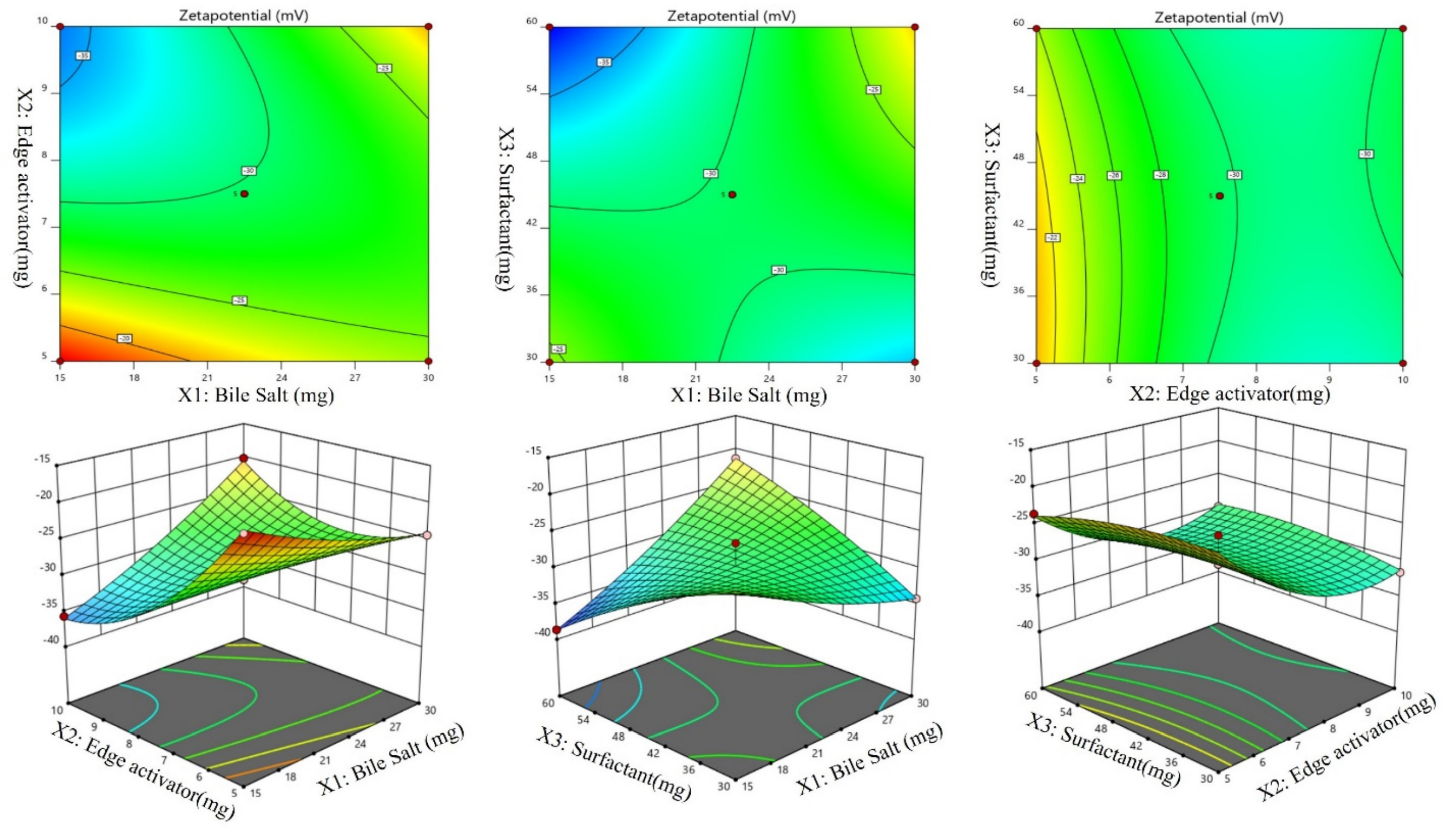
Effect of independent variables Bile salt (A), edge activator (B), and Span 60 (C) on ZP (Y_4_).

A Box-Behnken design was used to optimize the Dapa-BLs by taking different concentrations of bile salt (SDC, A), edge activator (Tween 80, B), and surfactant (Span 60, C). Their effects were determined by entrapment efficiency (Y1), Particle size (Y2), PDI (Y3), ZP (Y4) and as shown in Table 2. Linear, second-order, and quadratic models of the design space were used to analyze the response values. The design showed that the quadratic model fit the three variables the best. All the models were statistically examined, and the quadratic model was found to have the highest value (R^2^). ANOVAs on the quadratic model for each response showed that they were well-fit (p < 0.0001) and that the lack of fit was not significant (p > 0.05). Polynomial equations represented the responses, and the 3D response graph (Figures 2-4) revealed the independent variables' individual and combined effects on the replies. Figure 1-3 graphically represents all responses' actual and anticipated values.

### Effect of independent variables on entrapment efficiency (Y1)

The Dapa-BLs F1 formulation, prepared with the composition of 30 mg SDC, 7.5 mg tween 80, and 60 mg Span 60, had the lowest EE. The maximum EE (86.37±2.6%) was found to be for Dapa-BLs (F13), prepared with a composition of 15 mg bile salt, 7.5 mg edge activator, and 60 mg Span 60, as displayed in Table 2. The 3D response plot (Figure 1) showed that the entrapment effectiveness of Dapa decreased with increasing bile salt (SDC) concentration. In order to boost solubility, micelles can develop in the dispersion media, which could account for the fall in EE. In addition, a decrease in entrapment efficiency is shown as SDC concentration rises because of the fluidizing action on the lipid bilayer membrane 46, 47. In addition to SDC and Span 60, the edge activator hurt entrapment efficiency. Boosting the Span 60 concentration improved entrapment effectiveness. Span 60's extended alkyl chain and higher transition temperature increased EE 48,49. Below is the software-generated quadratic polynomial equation for entrapment efficiency.

Entrapment efficiency (Y1, %) = EE = +56.85 -09625A +1.04B +7.19C -4.99AB -4.98AC -4.98BC +7.70A^2^ +7.25B^2^ +1.93C^2^ (1)

### Effects of independent variables on particle size (Y2)

The particle size of the prepared Dapa-BLs formulations was found in the range of 155.36±2.48 nm (Dapa-BLs, F13) to 286.12±4.22 nm (Dapa-BLs, F11), as depicted in Table 2. Figure 2 is a 3D response surface plot illustrating the significant impact the applied variables had on particle size (A-F). Dapa-BLs F11's compact formula includes only 15 mg of bile salt, 7.5 mg edge activator, and 60 mg of Span (60 mg). The maximum size was shown by the Dapa-BLs F11 formulation, which contained 30 mg of bile salt, 7.5 mg of edge activator, and 30 mg of Span 60. The significant variations in the results showed that the used variables were important parameters for the optimization process. Over time, the particle size expanded in response to an increase in SDC. The bile salt imparted the negative charge on the surface of the BLs, and this charge increased with the increase in SDC concentration. The lipid bilayer enhanced particle size due to a higher repulsive force 50. Because SDC has such a hefty structure, the particle size has also grown larger 51. The second factor, edge activator, also showed a significant effect on the particle size. As the concentration increased, the particle size also increased due to the formation of a large shield and steric stabilization. With a greater concentration of hydrophilic polyethylene oxide residues, there was a greater capacity for water intake and consequent growth 52, 53. For the third factor, Span 60, the effect was negative; specifically, the solubility of Dapa rose as the particle size reduced because of a decrease in interfacial tension as the concentration of Span 60 increased. In order to calculate the hydrodynamic diameter, the following quadratic polynomial equation is used.

Particle Size (Y1) = +267.41 +14.15A -5.17B -37.16C +9.63AB -1.64AC +19.40BC -31.38A2 -50.06B2 -12.46C2 (2)

We can observe that the bile salt SDC, the edge activator Tween 80, and the surfactant Span 60 are all used as codes in the equation for the three independent variables A, B, and C. Significant (p≤ 0.05) effects were seen for the terms A, B, and C, as well as A, C, BC, A2, B2, and C2. The hydrodynamic diameter was affected both by these parameters separately and in combination. The positive sign in the equation represents an increase in the effect, whereas the negative sign represents a decrease in the effect. The lack of fit was nonsignificant (F = 1.98, p = 0.2596), indicating a quadratic model. The close values of the predicted R2 (0.7471) and the adjusted R2 (0.9420) were found to be in close agreement, as presented graphically in Figure 2 (A-F). The adequate precision was >4 (14.8427), indicating that the model had an acceptable signal.

### Effect of independent variables on PDI (Y3)

It can be seen in Table 2 that the PDIs of the developed Dapa-BLs formulations vary from 0.126 ±0.23 (Dapa-BLs F13) to 0.419 ±0.15 (Dapa-BLs F3). As seen in the graph of Figure 3, the used factors did have a noticeable impact on the PDI. The smallest PDI was observed with the Dapa-BLs F13 formulation, which included 15 mg bile salt, 7.5 mg edge activator, and 60 mg of Span 60. Maximum PDI was achieved with a combination of 45 mg Span 60, 7.5 mg edge activator, and 22.5 mg bile salt. A large range of outcomes indicated that the variables employed were critical optimization parameters. It was discovered that as the SDC was raised, the PDI value rose (A). A possible explanation is larger particle hydrodynamic diameters 55. The second factor, edge activator (B), showed an insignificant effect on the PDI. However, as the Span 60 (C) concentration increased, the PDI of the Dapa-BLs vesicles decreased due to the reduction in the hydrodynamic diameter. While the hydrodynamic diameter of Dapa-BLs particle dropped as The PDI of these particles increased as the concentration of Span 60 (C) remained rather constant. The following is the software-generated polynomial equation for PDI 56:

PDI (Y3) = +0.4116 +0.0034A -0.0282B -0.0506C -0.0240AB +0.0583AC +0.0685BC -0.1092A2 -0.0979B2 -0.0572C2 (3)

The model terms A2, B2, and C2 were significant (p< 0.05), but B was not (p > 0.05). According to the results, the quadratic model was significant (p> 0.05) with an F-value of 90.42. The poor fit did not affect the significance of the results (p > 0.05), which was excellent news for the model. Figure 3 displays that the projected R^2^ of 0.9520 and the adjusted R^2^ of 0.9805 agree reasonably well (A-F). The adequate precision was found to be >4 (24.61), revealing that the model had an adequate signal.

### Effect of the independent variables on zeta potential, (Y4, ZP)

According to the ANOVA results from Table 3, the model can describe well the effect of different independent variables on ZP response (p < .0001). Figures 4 (A-F), revealed that, ZP was significantly affected by changing bile salts (X1). Moreover, changing X1 concentration (X2) and X3 significantly influenced ZP (p < .0001). ZP value for Dapa-loaded BLs was significantly increased by increasing the bile salt concentration. These results were counter-intuitive and could be attributed to the bile salt anionic nature, as it would be expected that increasing its concentration could increase the negativity of the prepared vesicles. The Predicted R² of 0.9427 is in reasonable agreement with the Adjusted R² of 0.9478; i.e. the difference is less than 0.2. Adeq Precision measures the signal to noise ratio. A ratio greater than 4 is desirable. Your ratio of 21.100 indicates an adequate signal.

The Model F-value of 33.31 implies the model is significant. There is only a 0.01% chance that an F-value this large could occur due to noise. P-values less than 0.0500 indicate model terms are significant. In this case A, B, AB, AC, B² are significant model terms. Values greater than 0.1000 indicate the model terms are not significant. If there are many insignificant model terms (not counting those required to support hierarchy), model reduction may improve your model. The Lack of Fit F-value of 0.09 implies the Lack of Fit is not significant relative to the pure error. There is a 95.96% chance that a Lack of Fit F-value this large could occur due to noise.

### Validation of ondependent variables

The EE Predicted R² value of 0.8499 exhibits a high level of agreement with the Adjusted R² value of 0.9691, being within a margin of less than 0.2. Adeq Precision quantifies the ratio of the signal to the noise. Optimal ratio is one that exceeds 4. Based on the ratio of 26.845, the signal is considered sufficient. The particle size Predicted R² of 0.7650 is in close proximity to the Adjusted R² of 0.9443, exhibiting a magnitude difference of less than 0.2. Asymptotic Precision measures the signal-to-noise ratio. An optimal ratio is greater than 4. The signal is deemed adequate using a ratio of 15.370. Given that the difference between the PDI Predicted R² of 0.9520 and the Adjusted R² of 0.9805 is less than 0.2, the two are reasonably consistent. Asymptotic Precision measures the signal-to-noise ratio. An optimal ratio is greater than 4. A ratio of 24.619 indicates that the signal meet the required criteria. With a difference of less than 0.2, the zetapotential Predicted R² of 0.9427 is nearly equal to the Adjusted R² of 0.9478. Adeq Precision measures the quotient of the signal intensity to the degree of imposed noise. An optimal ratio is more than 4. An analysis of the ratio of 21.100 indicates that the signal is adequate.

### Selection of optimized formulation (Dapa-BLs opt)

**Figure 5:**
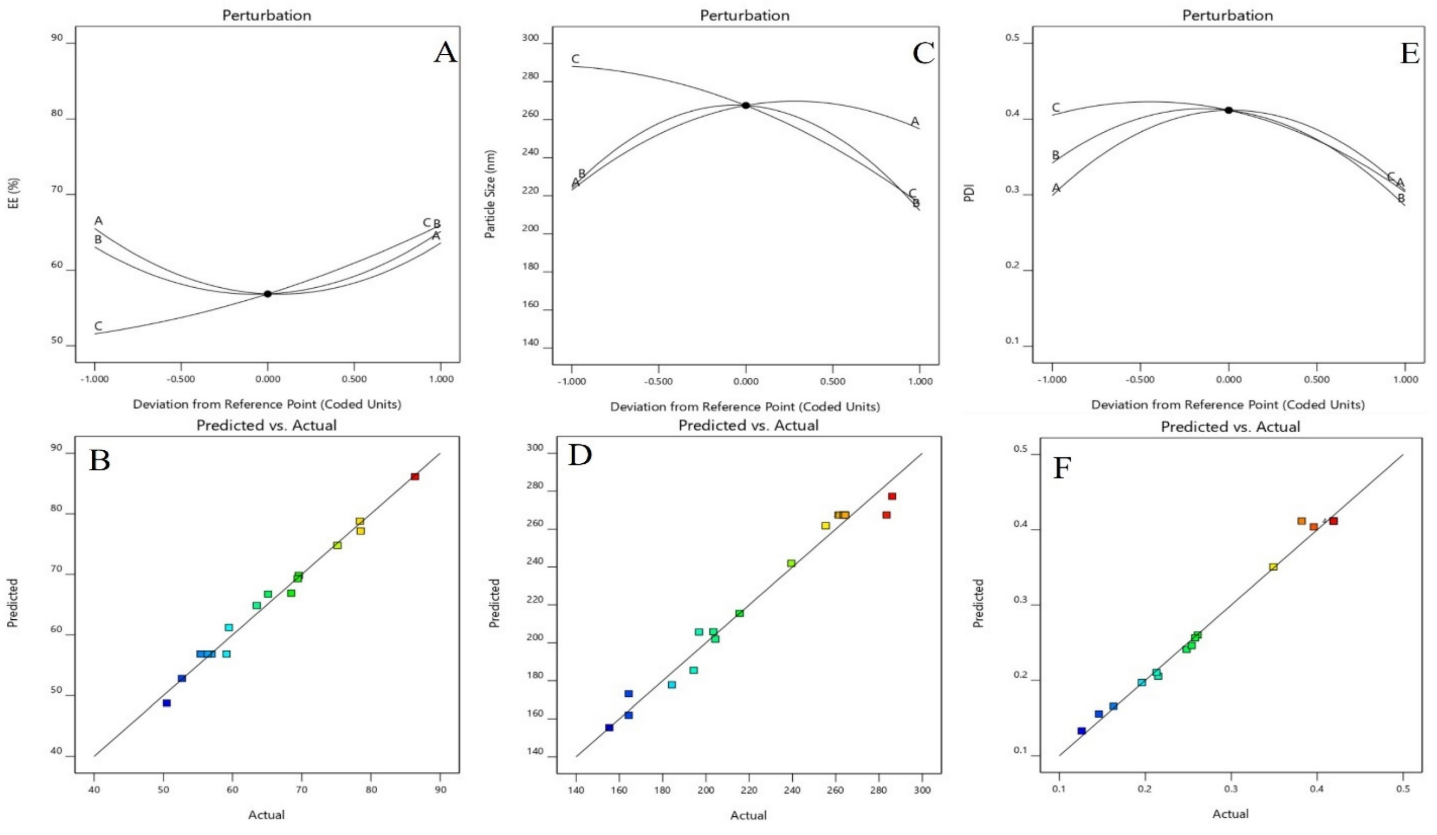
Perturbation plot and Actual and predicted images of (**A &B**) Particle size (Y_1_), (**C&D**) entrapment efficiency (Y_2_), (**E&F**) PDI.

**Figure 6:**
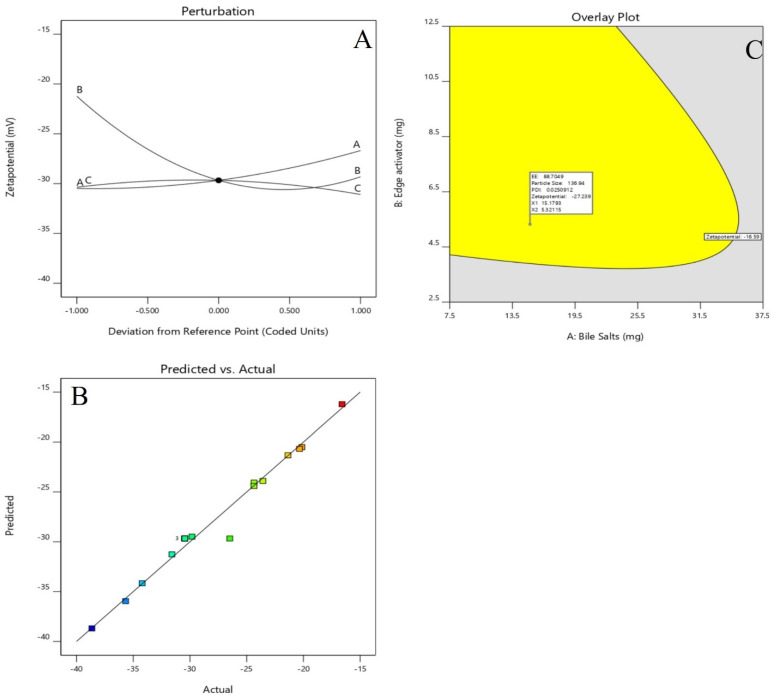
Perturbation plot and Actual and predicted images of (**A &B**) of Zeta Potential and Desirability plot of Optimized Dapa-BLs.

Point prediction was used to determine the best formulation (Dapa-BLs opt). A mixture of 15 mg SDC, 7.5 mg edge activator, and 60 mg Span 60 was used to create the optimal Dapa-BLs opt. The PDI was 0.126 ±0.23, and the ZP was -38.64 ±1.30; the entrapment efficiency was 86.37 ±2.6%; the hydrodynamic diameter was 155.36 ±2.48nm, and the ZP was -38.64 ±1.30 in experimental conditions. The program estimated a hydrodynamic diameter of 135.508nm, an entrapment effectiveness of 92.90%, a particle distribution index of 0.111, and a zeta potential of -42.76. As shown graphically in Figures 5 & 6 and the overlying plot, the anticipated and practical outcomes were in close agreement, indicating that the model was well-fit (4E). Using a digital pH meter, we observed that the ready-to-use Dapa-BLs had a pH of 6.3-6.5. The Dapa-BLs opt formulation was determined to contain a 10% drug loading. The desirability values of the optimized Dapa-BLs are listed in table 4.

### Particle size distribution and zetapotential

EE% was found in the range of 50.49±0.95 to 86.37±2.6% (Table 1). The average EE% for all 17 prepared formulations was found to be 61.79 ± 0.92%. Improved EE% was detected, likely as a consequence of the lipophilic chemical attraction of Dapa to the lipophilic area inside the lipid bilayers. SDC in the lipid bilayers was thought to be capable to solubilize and accommodate the drug. It was observed that there was a slight increase in EE% of Dapa in nano-bilosomes when the SDC concentration changed from 15 to 30mg. Although the increase in EE% is not significant. As the concentration of SDC was further increased to 22.5 to 30 mg, the EE% started to reduce (not significant), indicating that the ability of solubilization of drug in a lipid bilayer by SDC was limited, a similar outcome of EE% of hexamethylmelamine (lipophilic compound) was reported by the Sun *et al.* 2010 44. Based on the smallest vesicles size, PDI, comparable zeta potential, and EE%, formulation Dapa-BLs F13 was selected as an optimized formulation and characterized further for *in vitro* and *in vivo* activity.

### DSC studies

Differential scanning calorimetry (DSC) is a technique for measuring thermal changes in a material without any mass change. Because the exposure duration to the harsh condition was short in this experimental approach, it was difficult to detect a significant change in dapagliflozin, although the crystal form may be lost over long-term exposure, according to the reference literature. Dapagliflozin's surfactant thermogram indicated a pronounced endothermic peak at 151.900C (Figure 6), revealing its crystalline nature. The shift of the drug from crystalline nature to amorphous is indicated by a change in the thermal behavior of endothermic peaks of the optimized formulation. The thermogram of Cholesterol and SDC exhibited its characteristic peak at 61.48 °C and 325.64 °C, respectively (Fig. 6). The thermogram of Dapa-BLs opt showed only one characteristic endothermic peak at 82.37 °C (Fig. 4D), which is closer to the melting point of CHO. There was no other characteristic peak Dapa found in thermogram, which indicates that AG was completely encapsulated into a lipid bilayer matrix.

### Fourier Transform Infrared Spectroscopy Study (FTIR)

The Bilosome Approach to Formulation By comparing the fingerprint region (below 1500cm^-1^) of the improved formulation (F13) (Figure 9) with that of its components (Dapa, S60, SDC, and cholesterol), FTIR spectra assured bilosome particle production. FTIR analysis of Peaks in absorption was seen at 3367.10 cm^-1^ (OH stretching), 1613.16 cm^-1^ (C=C, aromatic), and 1246.70 cm^-1^ for pure Dapa (C-O ester stretching). Peaks at 1018 cm^-1^ for the C-Cl bond and 3375 cm^-1^ for the O-H elastic response were frequent from Dapa and 1614 cm^-1^ for the C-C bond. The O-H group was seen at 3330 cm^-1^, followed by alkanes and aromatic rings at 1449 cm^-1^. The bands at 2939 cm^-1^ (aliphatic C-H) and 1562 cm^-1^ (COO) are most associated with SDC. One can observe the O-H group of cholesterol at a frequency of 3341 cm^-1^, and the alkanes and aromatic rings can be seen at 1454 cm^-1^. There was no evidence of an in the Fourier transform infrared spectra; the drug and other formulation elements interact chemically with the drug or the optimized formulation F13. However, there is no overlap between fingerprint locations, providing more evidence that physical traits can shift.

### SEM image studies

**Figure 8:**
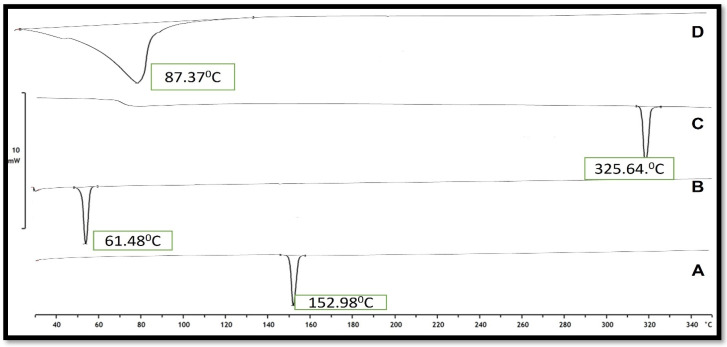
DSC thermogram of A) Pure Drug, B) Cholesterol, C) SDC and D) Optimized Dapa-BLs.

**Figure 9:**
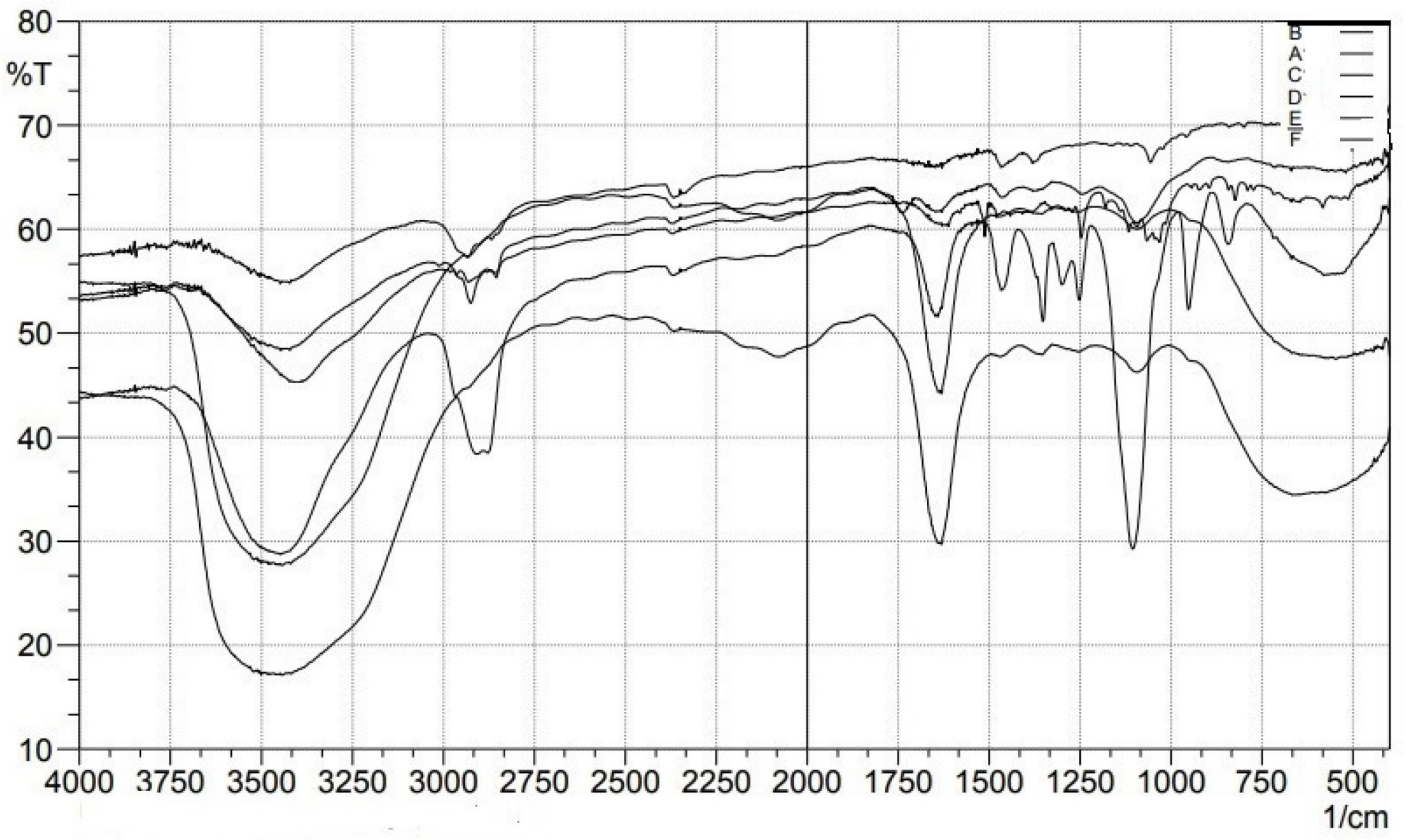
FTIR spectral studies of A) Pure Drug, B) Cholesterol, C) SDC, D) P123, D) Tween 80, E) Span 60 and F) Optimized Dapa-BLs.

SEM was used to examine the form and surface morphology of optimized Dapa-BLs, and the image revealed a spherical shape with smooth surfaces and no aggregation. As demonstrated in Figure 8, SEM describes the surface morphology of the drug and excipient 40. This nanometric size indicates that BLs can be absorbed by Peyer's patches and delivered to the intestinal lymphatic system without going through the liver, increasing the drug's oral bioavailability. Malvern's particle size measurement is somewhat more significant than that of SEM. This can be explained in the following way: the hydrodynamic size of a nanoparticle is assessed by differential light scattering, which is the size of the nanoparticle plus the liquid layer around it, whereas the size is determined by SEM, which is the actual size of the nanoparticle. For absorption through cells and into lymphatic tissue, round and smooth particles are frequently preferred 41.

### *In vitro* dissolution study

**Figure 11:**
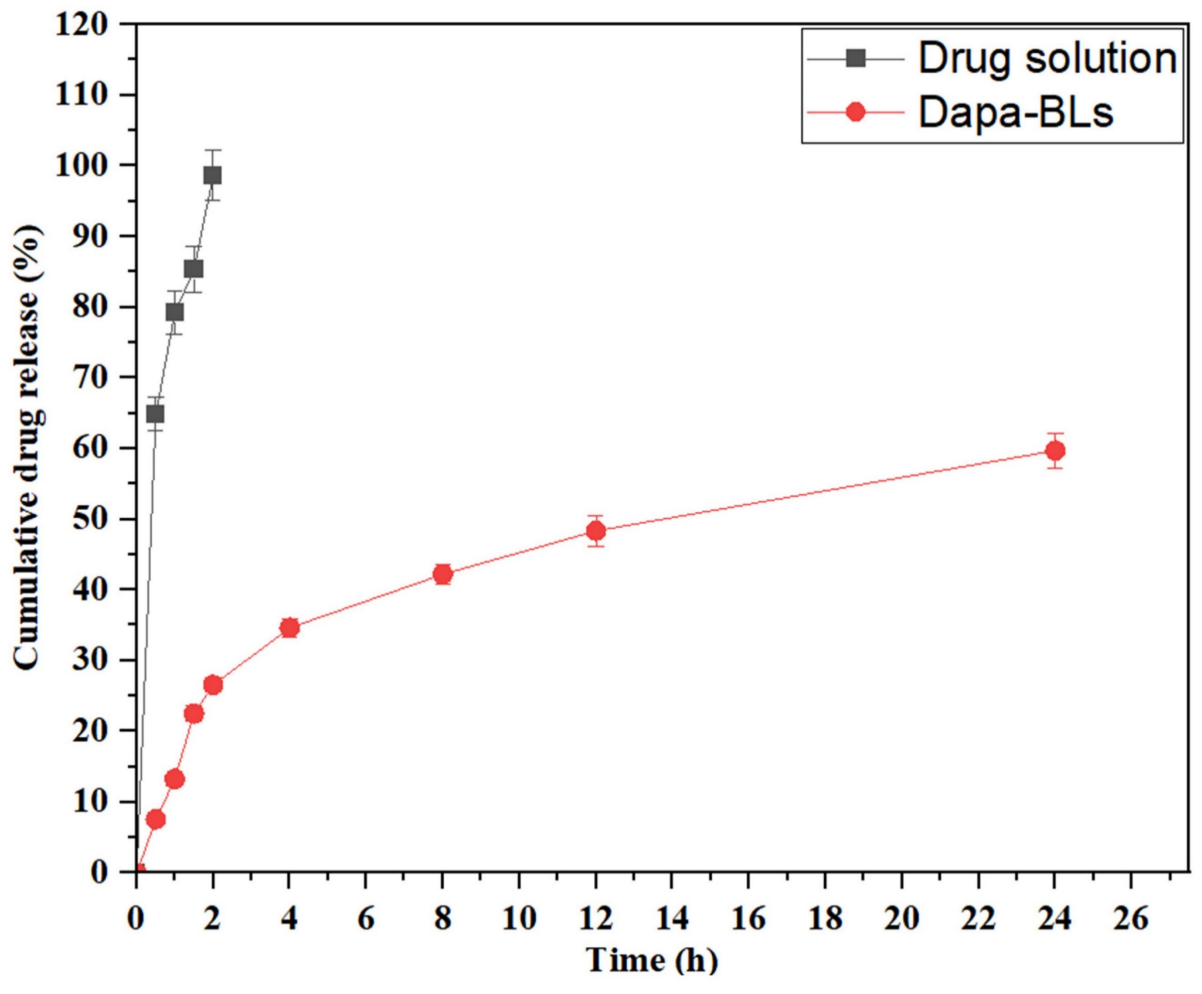
*In vitro* drug release study of optimized Dapa-BLs and Dapa-solution.

Dapa-BLs (F13) and pure Dapa solution drug release studies were performed using a hemodialysis bag, and the results are depicted in Figure 11. Through 24 hours of testing, we determined that the amount of Dapa released from our optimized Dapa-BLs was 59.68 ±1.23%. In contrast, the Dapa solution released 98.67±1.05% after only 2 hours. The drug release rate from optimized Dapa-BLs was much lower than that from the pure drug solution. The drug in this situation had to penetrate the bilosome bilayer and the polymer matrix. This allowed for a more gradual release of the drug. It also showed a bimodal release profile with a rapid onset and a gradual tail-off. When compared to the drug solution, Dapa-BLs showed much lower drug release. This was likely due to the Dapa-BLs' higher viscosity. The release mechanism was determined by calculating a variety of kinetic models. According to the results, the coefficient of determination (R^2^) for the zero-order kinetic model is 0.6938; for the first-order kinetic model, it is 0.8962; for the Higuchi model, it is 0.7964; for the Korsmeyer-Peppas model it is 0.9631, and for the Hixon-Crowell model it is 0.8537. According to the results, the Korsmeyer-Peppas model is the most appropriate kinetic release model based on the highest R2 value. Anomalous transport, i.e., diffusion-based drug release from optimized Dapa-BLs, was suggested by the exponent n = 0.58.

### *Ex vivo* permeability study

Rat gut was used to investigate the *ex vivo* permeability of both optimized DAPA-BLs and Dapa solution. Dapa-BLs had a drug permeation rate of 79.36 ± 2.16 g/cm^2^, which was statistically substantially greater (p< 0.05) than that of drug solution (20.68 ± 0.94 g/cm^2^). Dapa-BLs were shown to have a flux value of 1.53 g/cm^2^/h, 3.32 times higher than Dapa solution (0.46 g/cm^2^/h). It was also determined that the optimum values for the diffusion coefficients of Dapa-BLs and drug solution are 1.23 ×10.4 cm/min and 2.75 ×10.5 cm/min, respectively. Nanosized particles increased internalization in the lipid matrix, and the presence of nonionic surfactant all contributed to the enhanced penetration 58. As a result of increased hydrostatic pressure, the tight junction of the gut opens when a nonionic surfactant is present, allowing more water to pass through. In addition to decreasing reticuloendothelial absorption, the surfactant also blocked the Pgp efflux pump. Also, the inclusion of bile salt in a formulation reduces its efflux, and the bilosomes' particles' malleability is improved.

### Biological study

#### Pharmacokinetic study

**Figure 12:**
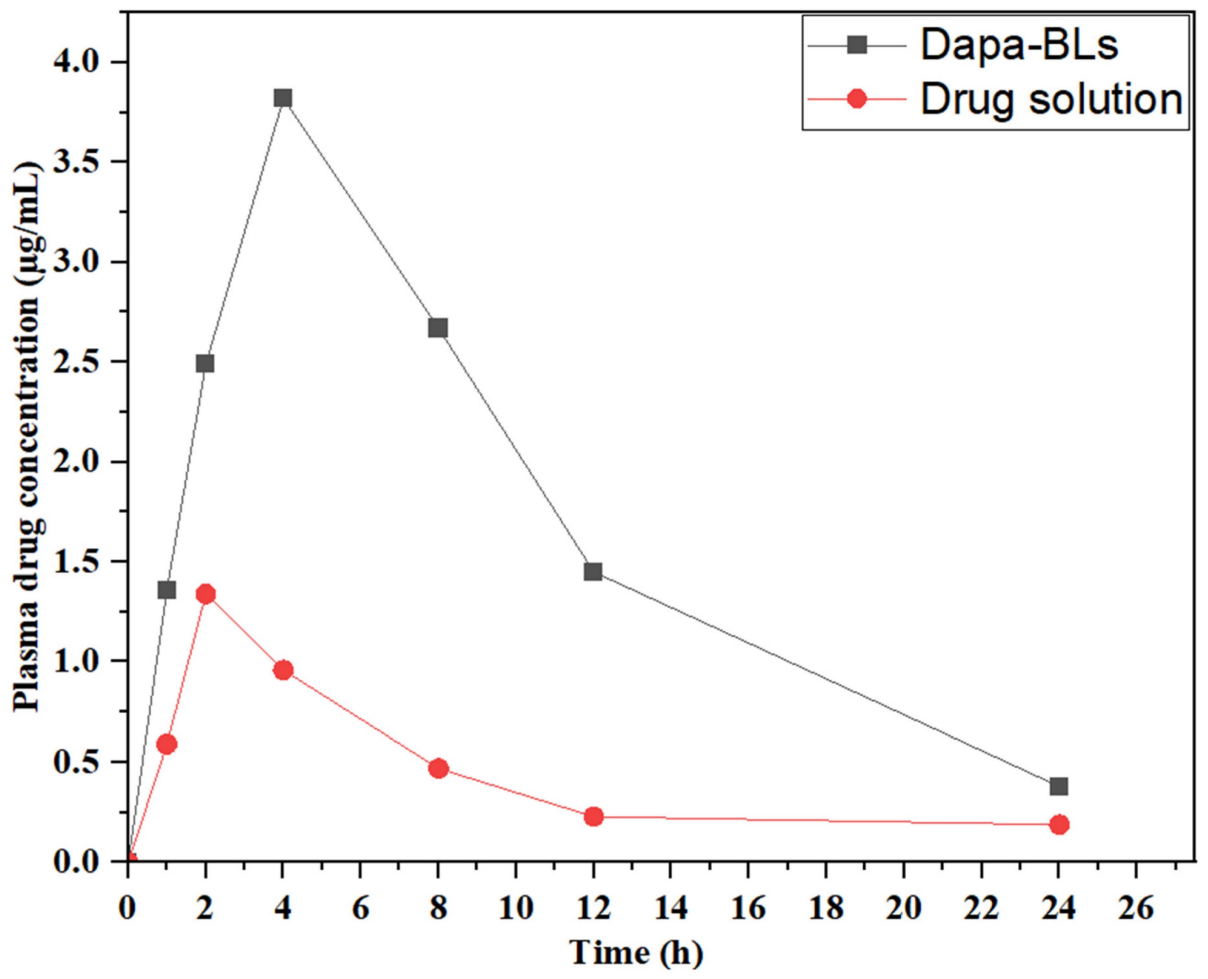
Dapa-BLs and drug solution plasma concentration vs. time graph in rats at oral administration.

Pharmacokinetic research was conducted using optimized Dapa-BLs and a drug solution to measure drug bioavailability. Figure 12 shows the time-dependent drug concentration profiles of the optimal Dapa-BLs and the drug solution. The optimized Dapa-BLs showed significantly higher (3.82 ± 0.13 µg/mL, *p*< 0.05) Cmax value than Dapa solution (1.36 ± 0.06 µg/mL). Due to their tiny, well-encapsulated, easily soluble in stomach acid, highly permeable, and avoidance of first-pass metabolism, optimized Dapa-BLs have higher C_max_. Rats given Dapa-BLs had an AUC_0-24_ of 49,528.136 (g. h/mL) and an AUC_0-∞_ of 4,960 ±342 (g. h/mL), respectively. Compared to the Dapa-solution (AUC_0-∞_ 2.496 ±0.36; AUC total 14.495 ±2.03), The results show that these values are considerably (*p*<0.05) higher (3.41 and 1.99-fold). Dapa-BLs likewise had a longer T_max_ (4.02 h) than Dapa solution (2.05 h). Dapa-BLs exhibited 285.34 ±8.31 g.h^2^ /mL, while the AUMC0-t of the drug solution was 59.34 ± 2.16 g.h2 /ml. Slow and extended release of Dapa contributes to maximum drug absorption, as reflected by the high values of AUC and T_max_. We calculated an elimination rate constant (h-1) for Dapa-BLs and a drug solution of 0.138 and 0.7776, respectively. The calculated half-lives (h) for Dapa solution and Dapa-BLs are 2.05 ±0.21 and 4.02 ±0.31. The overall results indicate Dapa-BLs enhance the relative bioavailability of ~ 3.41-fold as compare to free drug solution. The higher bioavailability was found due to the higher uptake of bilosomes by intestinal M-cell of Peyer's patch and also due to increased solubility in the presence of lipid and surfactant 59.

#### Evaluation of hypoglycemic activity

Average fasting blood glucose level (BGL) was measured to calculate the antihyperglycemic impact of Dapa-BLs and Dapa solution, as shown in Fig. 11. Both the normal and diabetic control groups of high amounts of glucose in the blood were seen in rats of 203 ± 2.6 mg/dL and 206 ±1.9 mg/dL, respectively. Data revealed that after 1 hour of treatment, blood glucose levels dropped and stayed low for up to 12 hours with Dapa-BLs and up to 4 hours with Dapa solution. Blood glucose levels were observed to be lowered by a maximum of 59.60% (121 ± 1.9 mg/dL in 12 h). Significantly better results were seen with Dapa-BLs than with the Dapa solution and the diabetes control group (*p* <0.001**, *p* <0.0001#). After 12 hours, a person's blood glucose level can rise to 153 mg/dL. After 4 hours, the drug solution was associated with a rise in BGL, which peaked at 201 2.5 mg/dL 24 hours later. Animals given Dapa-BLs demonstrated sustained reductions in blood glucose levels, suggesting that the compound's improved solubility is responsible 60.

#### Biochemical evaluation

**Figure 14:**
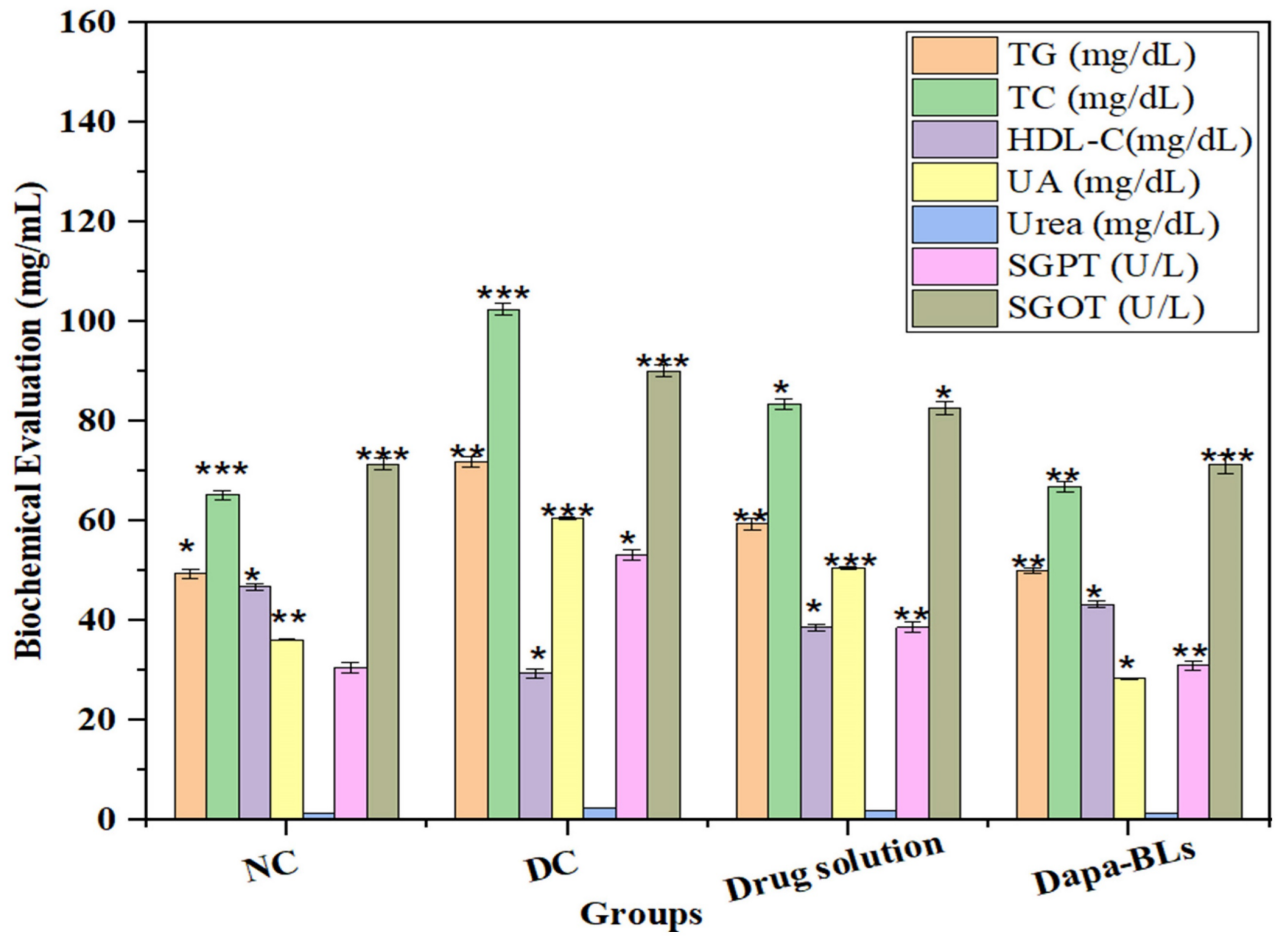
Estimated biochemical parameters from several treatment groups are compared. Data are shown as mean ± SD (n = 3). (* *p* < 0.001; ** *p* < 0.0003; *** p < 0.0001).

Serum levels of several biochemical indicators (including total cholesterol, high-density lipoprotein cholesterol, urea, serum glutamic-pyruvic transaminase (SGPT), uric acid, and serum glutamic oxaloacetic transaminase (SGOT), were measured at the study's conclusion. Figure 14 shows the Normal control, diabetes control, other biochemical parameters, a drug solution, and optimized Dapa-BLs. Rats with STZ-induced diabetes had significantly different blood glucose levels than control rats (*p* <0.0001). Type 2 diabetes is linked to cardiovascular problems 48, 49 because of the alteration in lipid profile. The STZ also causes liver toxicity due to alteration in serum glutamic-pyruvic transaminase and serum glutamic oxaloacetic. Related to diabetic control rats, the optimized Dapa-BLs and drug solution-treated group showed a significant (*p* <0.0001) decrease in increased TC and TG and a lowered level of HDL-C. In addition, the adjusted Dapa-BLs reduced the raised levels of uric acid, urea, serum glutamic-pyruvic transaminase, serum creatinine, and serum glutamic oxaloacetic transaminase when compared to diabetic control rats (*p* <0.0001). There was also a notable difference in the blood total protein level between the diabetic control group and the group that took Dapa-BLs, which resulted in a much lower total protein level 61.

Sophisticated formulations may be required for Dapa-BLs to ensure stability, solubility, and bioavailability. Achieving the ideal formulation that maintains both efficacy and safety can be challenging. An essential task is to determine the stability of Dapa-BLs under various storage conditions. Therapeutic effectiveness can be influenced by degradation byproducts or alterations in stability. The ADME (absorption, distribution, metabolism, and excretion) dynamics of Dapa-BLs may show significant differences between preclinical models and human subjects. Maintenance of consistent and predictable pharmacokinetics in humans is of utmost importance. Determining the optimal dosage that simultaneously minimizes toxicity can be a difficult task, especially when the effects of Dapa-BLs differ from those of Dapa. The observed efficacy in animal models may not always apply to people owing to differences in biology, progression of diseases, and therapy response. The financial requirements of developing and commercializing a new therapy are substantial. It is imperative to evaluate the cost-effectiveness of Dapa-BLs about existing therapies or alternative approaches. The adoption and reimbursement of Dapa-BLs may be affected by their need to manifest significant therapeutic benefits in comparison to existing therapies 62.

## Conclusion

The current study supports the use of Dapa-loaded BLs as a promising oral drug delivery system for diabetic treatment. Bilosomes were successfully formulated and evaluated as a novel drug delivery system that enhances its solubility, skin permeation, and bioavailability. 17 BLs formulations were prepared by thin film hydration method according to a Box Behnken design to determine the possible optimized formulation. All formulations were evaluated and showed acceptable EE, nano particle size, and stable vesicles confirmed by PDI, ZP results. In addition, the release profile showed a biphasic pattern, and the permeation profiles obtained showed a noticeable enhancement in the permeation flux of bilosomes over the unformulated drug.

The prepared Dapa-BLs have shown nano size range, high entrapment efficiency, spherical shape, and higher *in vitro* drug release up to 12 h. The thermal analysis study revealed that Dapa was encapsulated in a lipid matrix. The intestinal permeation study revealed that higher amount of drug permeated (*p* < 0.05) than free drug solution. A pharmacokinetic study showed that Dapa-BLs enhance the systemic bioavailability and residence time than free drug solutions. The pharmacodynamic study also revealed a significant (*p* < 0.05) enhancement in the hypoglycemic activity and biochemical parameters of free drug solution. Our findings concluded that oral Dapa-BLs were found to be a better treatment alternative for diabetes with improved therapeutic efficacy. Optimized Dapa-BLs (F13) was picked by design expert 17 numerical optimization, it is composed of Span 60 as a non-ionic surfactant, and 7.5 mg of SDC as a bile salt. The overall results of this study give a good interest towards bilosomes enhancement role in the active transdermal delivery of Dapagliflozin.

The use of bile salts in the formulation might enhance the solubility of the medicine, augment its absorption, and maybe selectively affect particular biological processes. Pharmacological permeability and absorption in the gastrointestinal tract are influenced by bile salts. In the absence of bile salts, liposomes may not benefit from the enhanced solubility and absorption that are usually provided by bile salts. Their performance would mostly depend on the formulation, geometry, and surface characteristics of the liposomes. One significant omission in the study is the lack of a comparison between DAPA-loaded bile lipids (BLs) and DAPA-loaded liposomes without bile salts. Conducting a direct comparison of these formulations would provide invaluable insights into the influence of bile salts on improving the delivery and efficacy of drugs. Hypothesise that the inclusion of bile salts improves the administration and efficacy of DAPA in rats. Therefore, it may be inferred that bile salts play a crucial role in enhancing the absorption and accessibility of the antibiotic. Conversely, if there is no significant difference, the heightened complexity of bile salts may not provide any more benefits.

## Figures and Tables

**Figure 7:**
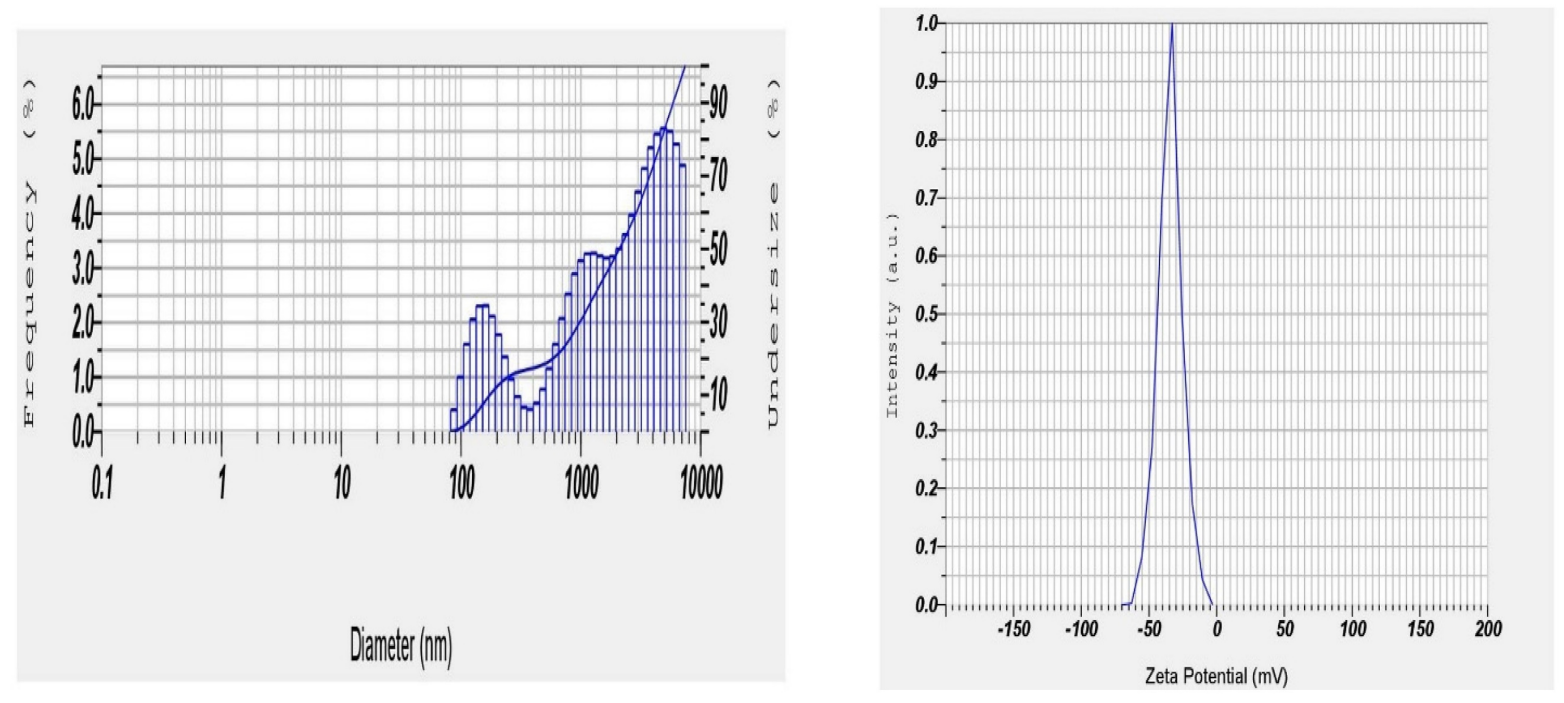
Optimized Dapa-BLs formulation particle size and Zetapotential (F13).

**Figure 10:**
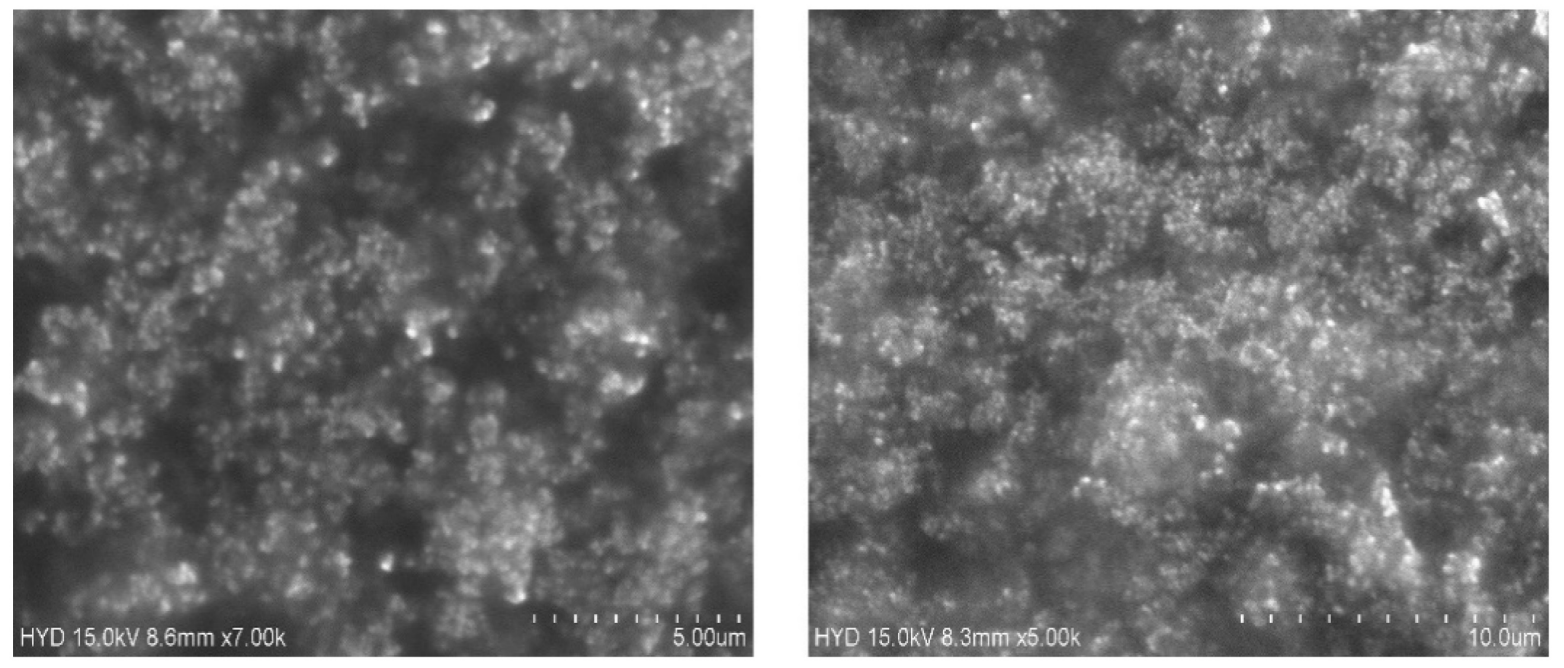
SEM images of Optimized Dapa-BLs with different intensity.

**Figure 13:**
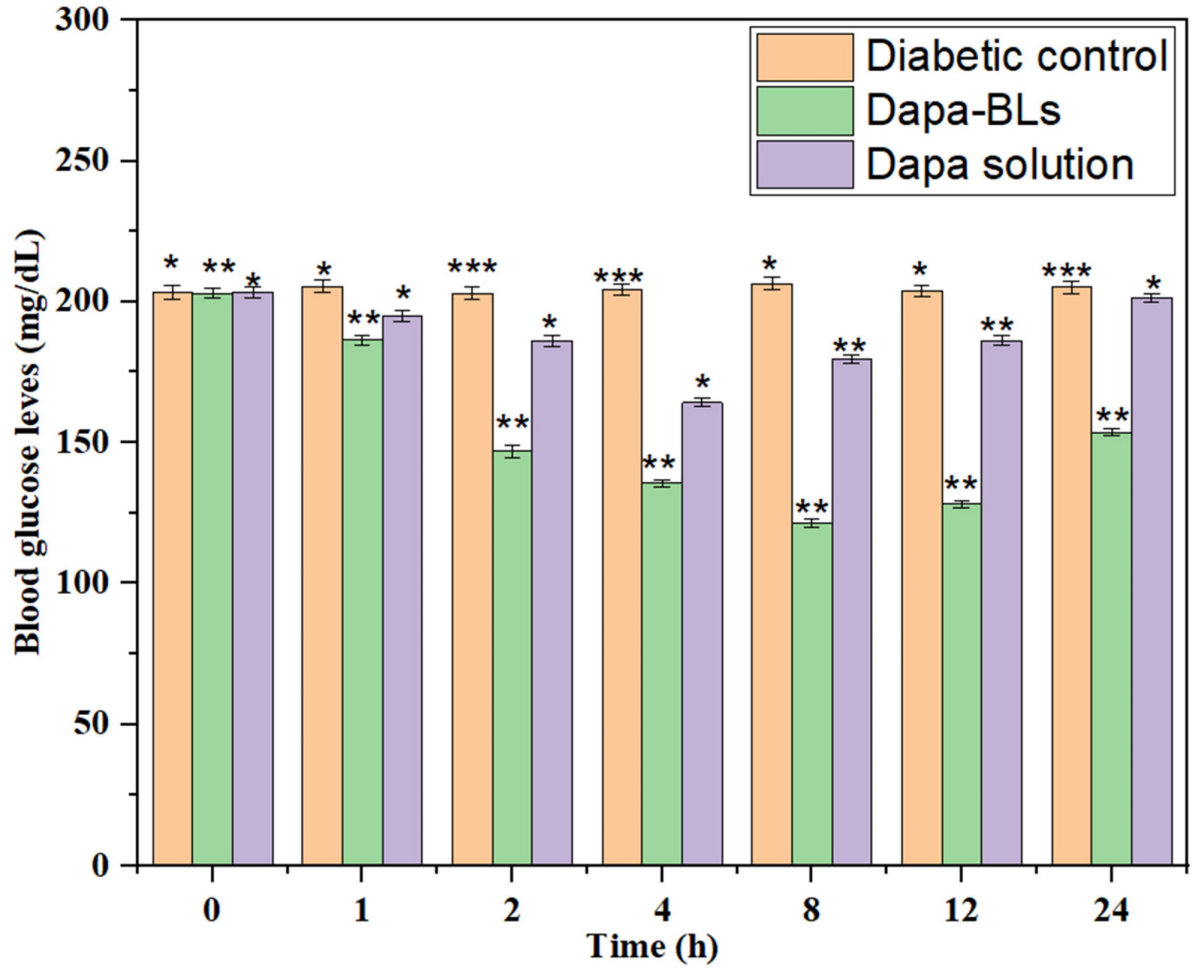
Results of Dapa-BLs and drug solution on mean fasting blood glucose levels over time. Data are shown as mean ± SD (n = 3). (*p < 0.001; **p < 0.0003; ***p < 0.0001).

**Table 1:** Box Behnken design variables and constraints.

Independent Variables					
Name	Low (-1)		Medium (0)		High (+1)
X1: Bile salts (mg)	50		65		80
X2: EA (mg)	15		22.5		30
X2: Surfactant	3		4		5
Dependent Variables					
Entrapment Efficiency (%)		50.49±0.95		86.37±2.6	
Particle Size (nm)		155.36±2.48		286.12±4.22	
PDI		0.126±0.23		0.419±0.15	
Zeta potential (ZP)		-16.59±1.64		-38.64±1.30	
					

**Table 2:** Independent variables and measured responses for the box Behnken design of Dapa loaded BLs.

Std	X1	X2	X3	Y1	Y2	Y3	Y4
1	30	7.5	30	50.49±0.95	239.48±2.51	0.349±0.02	-24.36±0.53
2	30	5	45	75.16±2.1	188.35±6.45	0.261±0.09	-24.35±1.26
3	22.5	7.5	45	56.34±0.64	264.35±3.98	0.419±0.15	-30.45±2.38
4	22.5	7.5	45	56.42±2.8	264.35±1.58	0.419±0.04	-30.45±1.52
5	22.5	7.5	45	55.39±3.1	263.52±3.46	0.419±0.06	-30.45±3.16
6	30	7.5	60	59.48±3.5	204.37±0.94	0.258±0.04	-21.36±0.49
7	15	10	45	78.41±2.5	164.32±2.43	0.196±0.26	-35.67±0.52
8	22.5	5	30	52.68±1.4	264.31±3.72	0.396±0.11	-20.34±0.43
9	22.5	10	60	69.42±2.9	184.26±6.24	0.254±0.14	-29.82±2.38
10	22.5	10	30	63.49±1.8	215.61±3.16	0.213±0.03	-31.59±1.05
11	30	7.5	30	69.58±2.4	286.12±4.22	0.248±0.05	-34.21±0.59
12	22.5	5	60	78.52±3.5	155.36±2.48	0.163±0.07	-23.57±0.52
**13**	**15**	**7.5**	**60**	**86.37±2.6**	**164.29±3.46**	**0.126±0.23**	**-38.64±1.30**
14	22.5	7.5	45	59.13±2.1	261.35±2.49	0.419±0.14	-30.49±1.08
15	30	10	45	68.49±2.2	196.83±3.75	0.146±0.16	-20.13±2.04
16	15	5	45	65.14±0.91	194.35±3.61	0.215±0.28	-16.59±1.64
17	22.5	7.5	45	56.99±3.7	283.49±3.64	0.382±0.41	-26.49±1.89

**Table 3:** Summary statistics for all expected responses using statistical design software for each model.

Parameter	Source	DF	Sum of squares	Mean of squares	F Value	*P*-Value
%EE	Model	9	1686.10	187.34	56.85	< 0.0001
	Residual	7	23.07	3.30		
	Lack of fit	3	15.28	5.09	2.61	0.1882
	Pure error	4	7.79	1.95		
SIZE	Model	9	31409.58	3489.95	29.89	< 0.0001
	Residual	7	817.30	116.76		
	Lack of fit	3	488.15	162.76	5.60	0.2596
	Pure error	4	329.15	82.29		
PDI	Model	9	0.1772	0.0197	90.42	< 0.0001
	Residual	7	0.0015	0.0002		
	Lack of fit	3	0.0004	0.0001		0.6899
	Pure error	4	0.0011	0.0003		
Zetapotential	Model	9	577.97	64.22	33.31	< 0.0001
	Residual	7	13.50	1.93		
	Lack of fit	3	0.8866	0.2955	0.0937	0.9596
	Pure error	4	12.61	3.15		

**Table 4:** Optimized formulation based on observed values of response and predicted value response.

Variables	Optimum composition	Response	Observed value of response	Predicted value of response	Percentage Error
X1 (mg)	15.179	Y1	86.37±2.6	88.703	0.973
X2 (mg)	5.321	Y2	164.29±3.46	156.951	1.046
X3 (rpm)	59.172	Y3	0.126±0.23	0.125	1.008
		Y4	-38.64±1.30	-35.246	1.096
Desirability: 0.926					
